# Traumatic Urethral Pseudoaneurysm in a 12-Year-Old Boy Leading to Progressive Urethral Stricture and Complete Obstruction: A Case Report and Literature Review

**DOI:** 10.7759/cureus.101713

**Published:** 2026-01-16

**Authors:** Shinsuke Yoshizawa, Kensuke Ohashi, Keita Kogure, Takahiro Hosokawa

**Affiliations:** 1 Department of Pediatric Urology, Saitama Children's Medical Center, Saitama, JPN; 2 Department of Radiology, Saitama Children's Medical Center, Saitama, JPN

**Keywords:** pediatric urethrorrhagia, post-traumatic urethral injury, transcatheter arterial embolization, traumatic urethral injury, urethral pseudoaneurysm, urethral stricture

## Abstract

Urethral pseudoaneurysm is an extremely rare cause of urethrorrhagia in children. Most previously reported pediatric cases required transcatheter arterial embolization and generally resolved without long-term sequelae. We present a 12-year-old boy who sustained blunt perineal trauma and subsequently developed a bulbar urethral pseudoaneurysm requiring two sessions of arterial embolization. Unlike prior reports, the patient developed progressive circumferential urethral stricture leading to complete obstruction five months after initial injury. To our knowledge, this is the first reported pediatric case in which a traumatic urethral pseudoaneurysm resulted in progressive urethral stricture and complete obstruction. The case highlights the importance of long-term follow-up in children with vascular urethral trauma.

## Introduction

Urethrorrhagia in children is uncommon and most frequently results from minor urethral trauma, typically seen after straddle injuries. However, when bleeding is excessive, persistent, or recurrent, rare etiologies such as traumatic urethral pseudoaneurysm must be considered.

Pseudoaneurysms of the internal pudendal artery or its branches have been described in only a handful of pediatric cases worldwide [1-4]. Reported mechanisms include straddle injury, bicycle accidents, catheter-related trauma, and iatrogenic urethrotomy. Despite severe initial bleeding, previously reported pediatric cases generally demonstrated good outcomes after selective arterial embolization, with no long-term urethral complications [1-4].

Urethral stricture in children, in contrast, is most commonly attributed to traumatic or iatrogenic injury, including instrumentation-related mucosal damage, and its pathogenesis is often multifactorial. Whether vascular urethral injury, endoscopic manipulation, and subsequent ischemic or inflammatory changes may coexist and contribute to later stricture formation remains incompletely understood.

We describe what appears to be the first pediatric case of traumatic urethral pseudoaneurysm complicated by progressive circumferential urethral stricture, ultimately leading to complete obstruction. Rather than implying a single causal pathway, we discuss this case from the perspective of concurrent and potentially multifactorial complications of trauma, and review the existing pediatric and adult literature.

## Case presentation

**Figure 1:**
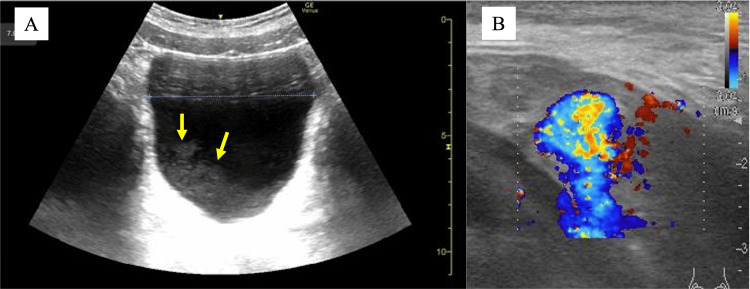
Abdominal ultrasound findings. (A) Abdominal ultrasound demonstrating intravesical blood clots (the yellow arrow indicates the blood clot). (B) Doppler ultrasonography demonstrating arterial flow within an intraluminal mass in the bulbar urethra.

A 12-year-old boy presented after being struck in the genital area by a ball, followed later that day by a fall from playground equipment, landing on his buttocks. He developed gross hematuria with blood clots that evening and presented to the emergency department. Vital signs were stable. Physical examination revealed penile ecchymosis and continuous venous bleeding from the external urethral meatus. Ultrasound imaging showed intravesical clots (Figure 1A), but the urethra was poorly visualized. Progressive urinary retention developed, and emergency cystoscopy under general anesthesia was performed.

Initial endoscopic findings

**Figure 2:**
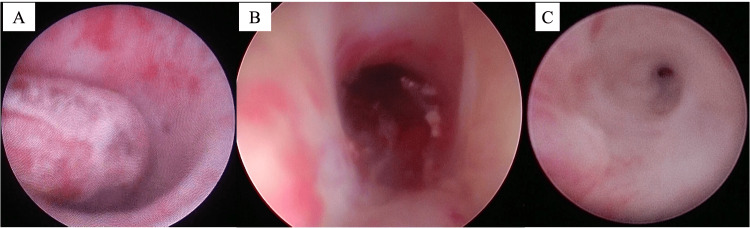
Serial cystoscopic findings. (A) Initial cystoscopic view showing a circumferentially bulging intraluminal lesion in the bulbar urethra. (B) Three weeks after injury, cystoscopy reveals resolution of the mass with mucosal defect and ulceration at the 6 o’clock position. (C) Five months after injury, cystoscopy demonstrates complete urethral obstruction due to progressive circumferential stricture.

Cystoscopy revealed a circumferential bulging lesion at the bulbar urethra completely obstructing the lumen (Figure 2A). The urethral mucosa was denuded between the 2-10 o’clock positions. A guidewire was successfully passed through a narrow 0-2 o’clock channel, allowing placement of a 14 Fr renal pelvis balloon catheter. The patient was diagnosed with traumatic urethral injury and discharged the following day.

Identification of urethral pseudoaneurysm

**Figure 3:**
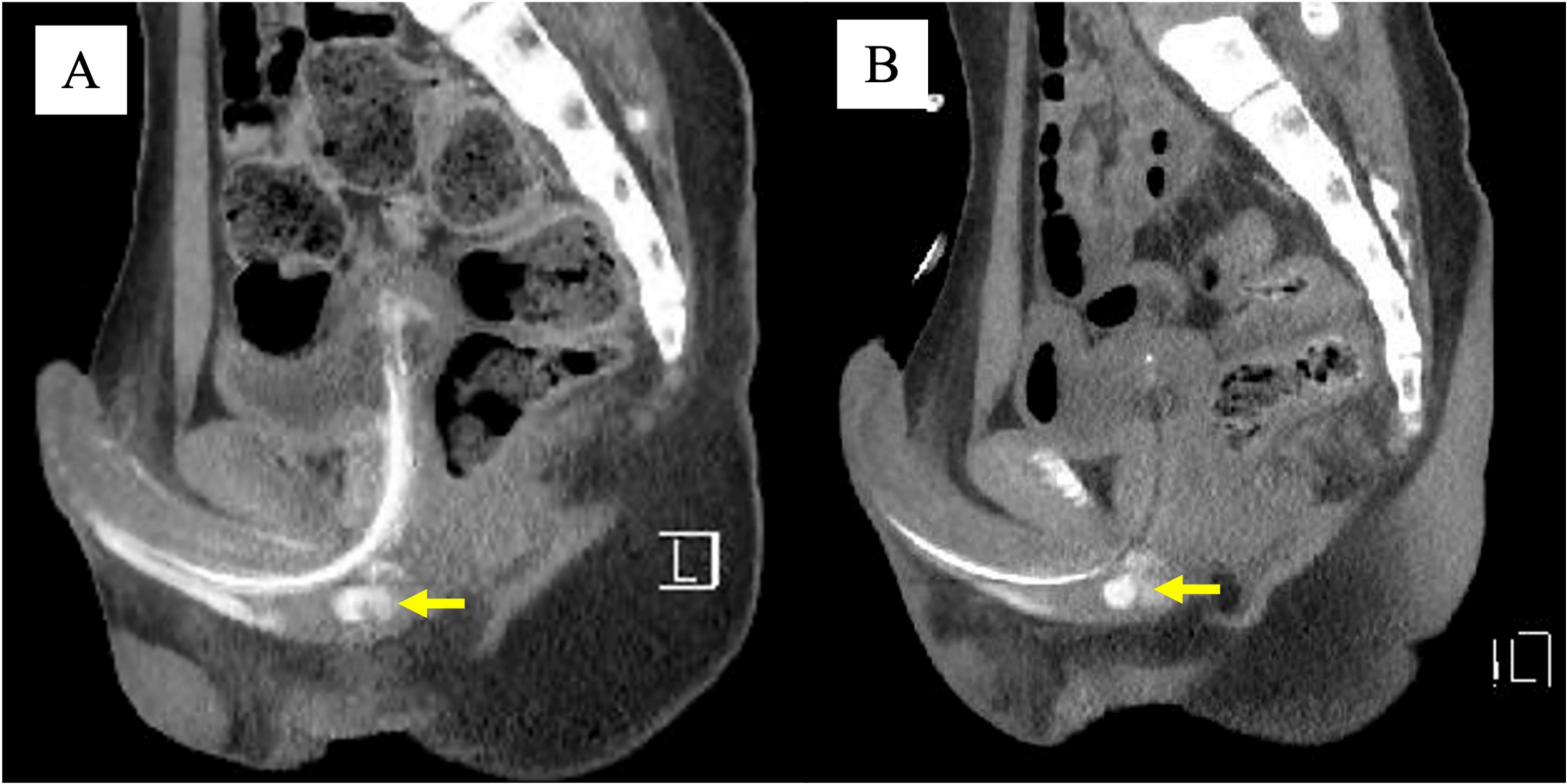
Contrast-enhanced CT findings of traumatic urethral pseudoaneurysm. (A) Contrast-enhanced CT showing contrast extravasation from the distal bulbar artery (the yellow arrow indicates the urethral pseudoaneurysm). (B) Contrast-enhanced CT obtained two days after transcatheter arterial embolization demonstrating recanalization (the yellow arrow indicates the urethral pseudoaneurysm).

**Figure 4:**
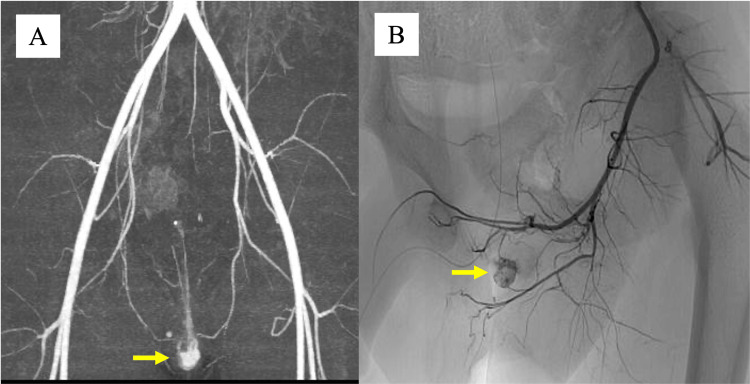
Angiography findings of traumatic urethral pseudoaneurysm. (A) Pre-embolization selective angiography of the internal pudendal artery demonstrating a pseudoaneurysm arising from the bulbar artery (the yellow arrow indicates the urethral pseudoaneurysm). (B) Pre-embolization angiography obtained from a different projection, clearly delineating the feeding branch and pseudoaneurysm (the yellow arrow indicates the urethral pseudoaneurysm).

After discharge, persistent minor urethral bleeding continued at home. On postoperative day 4, ultrasound demonstrated a vascularized mass in the urethra (Figure 1B). Contrast-enhanced CT confirmed a pseudoaneurysm at the distal bulbar artery (Figure 3A). Pelvic angiography performed via the right femoral artery demonstrated a pseudoaneurysm in the distal branches of the internal pudendal artery (Figure 4A, 4B). Selective embolization with Gelfoam was performed. Repeat CT two days later demonstrated recanalization (Figure 3B). A second superselective embolization of both bulbar artery branches was performed, after which no further perfusion of the pseudoaneurysm was seen.

Subsequent course

At three weeks, cystoscopy showed complete resolution of the intraluminal mass. A mucosal defect and ulceration at the 6 o’clock position were noted, consistent with healing of the pseudoaneurysm rupture site (Figure 2B). Continued catheter drainage was chosen to allow mucosal healing. At one month, although the mucosal defect had improved, a circumferential stricture proximal to the lesion had formed. A 17 Fr cystoscope could not be advanced, while a 13 Fr scope passed smoothly. As partial patency remained, the urethral catheter was removed, but the patient continued to experience voiding difficulty. Two weeks later, cystoscopy showed persistent circumferential narrowing; however, the scope was still passable. To protect urethral function and assess sphincter activity, a 10 Fr suprapubic catheter was placed, and the urethral catheter was removed. Despite diversion, the stricture progressed. At five months post-injury, cystoscopy demonstrated complete urethral obstruction (Figure 2C). 

Definitive repair was performed using an end-to-end urethral anastomosis. Cystoscopy demonstrated a circumferential urethral stricture with only a few-millimeter residual lumen. A 0.025-inch guidewire and a 4-Fr open-end catheter were successfully passed through the stricture.

An inverted-Y perineal incision was made directly over the stricture, and the bulbospongiosus muscle was split to expose the urethra. The stenotic segment appeared whitish and firm and was excised completely. The urethral defect measured 16 mm, and primary end-to-end anastomosis was deemed feasible. The proximal and distal urethral ends were mobilized, and an interrupted anastomosis was performed using 3-0 PDS sutures.

Postoperatively, a 16-Fr Foley catheter was left in place for one month and removed in the outpatient clinic. After catheter removal, the patient voided well, and the early postoperative course was favorable.

Informed consent

Informed consent for treatment and open-access publication was obtained from the patient’s legal guardian. All identifying information has been removed.

## Discussion

Traumatic urethral pseudoaneurysm is an exceptionally rare condition in children, with only a limited number of cases reported in the literature. Most pediatric cases result from blunt perineal trauma, straddle injuries, or catheter-related urethral injury. Reported mechanisms include sports-related blunt trauma, bicycle straddle injuries, traumatic catheterization, and injury during catheter removal. Adult case series similarly describe iatrogenic or instrumentation-related causes [1-5].

In the present case, the patient sustained two distinct perineal impacts on the same day. This “dual-impact” mechanism has not been previously emphasized in pediatric reports and may have contributed to more extensive arterial wall damage, predisposing to pseudoaneurysm formation.

Because urethral pseudoaneurysm clinically resembles more common causes of urethrorrhagia, diagnosis is often delayed. Typical findings include gross urethrorrhagia, intraluminal filling defects, and vascular flow on Doppler ultrasound. Selective angiography remains the diagnostic gold standard [1-5]. In our patient, Doppler ultrasound played a key role in raising early suspicion, consistent with prior pediatric reports [2,4].

Selective arterial embolization is widely accepted as the treatment of choice for urethral pseudoaneurysm. Various embolic materials, including Gelfoam, microcoils, and cyanoacrylate glue, have been successfully used, and bilateral embolization may be required in cases with significant collateral flow [2,3,5,6]. Although early recanalization is occasionally reported in adult cases, it is rarely described in children. Our patient required repeat embolization due to early recanalization. Gelfoam, as a temporary embolic agent, carries a risk of recanalization and repeated ischemia-reperfusion injury, which may contribute to prolonged local inflammation.

Importantly, all previously reported pediatric cases demonstrated favorable outcomes without long-term urethral sequelae [1-4]. By contrast, our patient developed progressive circumferential urethral stricture leading to complete obstruction. To our knowledge, this represents the first reported pediatric case of this outcome.

Several mechanisms may explain this progression, and a multifactorial etiology is most plausible. First, the initial trauma itself is a well-established cause of pediatric urethral stricture. Second, diagnostic cystoscopy and urethral instrumentation may produce mucosal injury and subsequent fibrotic healing; in our case, early cystoscopy demonstrated extensive mucosal denudation, which could predispose to stricture formation [7]. Third, vascular factors may have contributed: pseudoaneurysm formation and subsequent embolization, although selective, may have reduced local blood supply and exacerbated ischemic injury [5]. Accordingly, the circumferential cicatricial stricture observed in this patient is best interpreted as the result of concurrent and potentially multifactorial processes, in which the initial traumatic insult and possible iatrogenic effects of instrumentation likely played primary roles, with ischemic-inflammatory changes related to the pseudoaneurysm and embolization acting as contributing rather than definitive causes. Notably, embolization was performed under angiographic guidance using a selective approach, making inadvertent proximal arterial occlusion unlikely.

Additional factors may also have played a role. Circumferential compression by the intraluminal pseudoaneurysm could have promoted pressure necrosis. Pediatric tissues may exhibit robust inflammatory and fibroblastic responses, potentially accelerating scar formation [3,8]. Ongoing tissue growth and remodeling may further increase vulnerability to ischemic and inflammatory injury. Finally, the need for repeat embolization suggests significant collateral flow, which may have prolonged local inflammation and impaired healing [2,5].

From a clinical perspective, this case highlights several important considerations. Long-term follow-up is essential in children with urethral vascular injury, as delayed strictures may develop months after initial stabilization. Periodic cystoscopic surveillance should be considered even after successful embolization. Early suprapubic urinary diversion may help reduce ongoing urethral ischemia and mechanical stress in selected cases [7]. Taken together, this case suggests that even technically appropriate and selective embolization may not fully prevent delayed urethral complications when superimposed on severe trauma and possible instrumentation-related injury. Awareness of this risk may prompt earlier diversion and closer endoscopic surveillance in similar pediatric cases.

## Conclusions

We report a rare pediatric case of traumatic urethral pseudoaneurysm requiring repeated embolization and complicated by progressive circumferential urethral stricture leading to complete obstruction. This outcome has not been previously described in children. Importantly, based on a single case, a definitive causal relationship cannot be established. Rather, our findings support a concurrent and multifactorial process, in which severe initial trauma, potential iatrogenic effects of cystoscopic instrumentation, and vascular-ischemic and inflammatory mechanisms may all have contributed to stricture formation. Long-term follow-up is essential in pediatric patients with urethral vascular injuries, even after successful embolization, to monitor for delayed urethral complications. At the time of manuscript submission, postoperative follow-up is ongoing, and continued surveillance is planned to assess the durability of the surgical outcome.
